# Current Controversies in Newer Therapies to Treat Birth Asphyxia

**DOI:** 10.1155/2011/848413

**Published:** 2011-11-17

**Authors:** Pia Wintermark

**Affiliations:** Division of Newborn Medicine, Montreal Children's Hospital, McGill University, 2300 Rue Tupper, C-920, Montreal, QC, Canada H3H 1P3

## Abstract

Despite major advances in monitoring technology and knowledge of fetal and neonatal pathophysiology, neonatal hypoxic-ischemic encephalopathy (HIE) remains one of the main causes of severe adverse neurological outcome in children. Until recently, there were no therapies other than supportive measures. Over the past several years, mild hypothermia has been proven to be safe to treat HIE. Unfortunately, this neuroprotective strategy seems efficient in preventing brain injury in some asphyxiated newborns, but not in all of them. Thus, there is increasing interest to rapidly understand how to refine hypothermia therapy and add neuroprotective or neurorestorative strategies. Several promising newer treatments to treat birth asphyxia and prevent its devastating neurological consequences are currently being tested. In this paper, the physiopathology behind HIE, the currently available treatment, the potential alternatives, and the next steps before implementation of these other treatments are reviewed.

## 1. Introduction

Hypoxic-ischemic encephalopathy (HIE) is a serious brain injury due to birth asphyxia [1, 2]. HIE has a variety of perinatal causes of interrupted uterine and fetal blood flow and/or hypoxia, such as uterine rupture, placental abruption, and cord prolapse. However, most often, the exact timing and underlying cause remain unknown [3–5]. Its severity is also variable. Despite major advances in monitoring technology and knowledge of fetal and neonatal pathophysiology, birth asphyxia remains a serious condition that causes significant mortality and long-term morbidity. Causing 23% of all neonatal deaths worldwide [6, 7], it is one of the top 20 leading causes of burden of disease in all age groups (in terms of disability life adjusted years) by the World Health Organization [7]. Most deaths occur in the first week of life due to multiple organ failure or withdrawal of care due to the prospect of severe neurological impairments [8]. Moreover, children who survive birth asphyxia develop problems such as cerebral palsy, mental retardation, learning difficulties, and other disabilities [9]. 

Until recently, there were no therapies other than supportive measures for perinatal HIE. In the past several years, studies have been carried out to address the safety and efficacy of cooling in HIE [10–18]. Unfortunately, this neuroprotective strategy seems efficient in preventing brain injury in some asphyxiated newborns, but not in all of them. Thus, there is an increasing interest to rapidly understand the degree of brain injury and to possibly refine individual neuroprotective strategies or add neurorestorative strategies [19–23]. In this paper, the physiopathology behind HIE, the current available treatment, the potential alternatives, and the next steps before implementation of these other treatments are reviewed.

## 2. What Is Hypoxic-Ischemic Encephalopathy?

Neonatal brain hypoxic-ischemic injury is an evolving process initiated by the hypoxic-ischemic insult leading to decreased blood flow to the brain (primary lesions), followed by the restoration of blood flow in an injured brain and the initiation of a cascade of pathways (secondary lesions, “reperfusion injury”). This cascade includes accumulation of extracellular glutamate with excessive activation of glutamate receptors, calcium influx, and generation of reactive oxygen and nitrogen species, leading to cell death and definitive brain injuries [24]. This cascade is a primary target for neuroprotective interventions, but such interventions are currently limited by insufficient knowledge of the timing and duration of the so-called therapeutic window in newborns [19, 21, 22].

## 3. What Is the Current Available Treatment?

As the only neuroprotective treatment to have been clinically tested in large randomized controlled trials to minimize brain injury in asphyxiated term newborns [10, 11, 13, 14, 17, 18], cooling is thought to be protective in newborns (Table 1) mainly by reducing brain perfusion and metabolism, as well as mitigating reperfusion injury, reducing excessive free radical production, depressing the immune response and various potentially harmful proinflammatory reactions, and suppress epileptic activity [25]. Some results also suggest that moderate hypothermia can prevent or delay apoptosis and might be able to extend the therapeutic window for other interventions [25]. Results of these clinical trials in newborns have shown that moderate hypothermia after birth asphyxia reduces the risk of death and disability at 18 months, with a number needed to treat of nine asphyxiated newborns to prevent brain injury in one of them [12, 15, 16]. There was no real difference in any of the outcomes between whole-body cooling versus selective head cooling [12, 15, 16]. Only little adverse affects have been observed with this therapy; the main ones were arrhythmia and thrombocytopenia [12, 15, 16]. However, it remains unclear why induced hypothermia seems effective in preventing brain injury and improving neurological outcome in some asphyxiated newborns (mainly those with moderate HIE), but not in others (mainly those with severe HIE) [11, 14]. A better understanding of why brain injury continues to develop despite hypothermia treatment in some asphyxiated newborns is required in order to create and test some more specifically adjunctive therapies. Furthermore, early identification of patients who will develop further brain injury despite this treatment would be of utmost importance in order to determine those who would benefit most from adjustments in their hypothermia therapy or from adjunctive neuroprotective or neurorestorative therapies [20, 23].

According to the trials described above, mild hypothermia to an esophageal temperature of 33.5°C must be started in an asphyxiated newborn within 6 hours of life and continued for 72 hours. This temperature and this timing are based on animal data; for example the 6-hour limit for cooling initiation comes from data suggesting that the effectiveness of cooling diminishes as time increases from the hypoxic-ischemic event to initiation of cooling, with the closing of the “window” at a time between 5.5 and 8 hours after the event [26]. However, as previously mentioned, the exact timing of the “therapeutic window” is not known in human newborns. Some authors are thus starting to suggest that there might be a benefit from later initiation of cooling and increased depth or duration of cooling, as well as prolonged rewarming strategies [19]. Determination of the exact timing of the “therapeutic window” in asphyxiated newborns needs to be further investigated [27]. Additional randomized controlled trials are required to test if adjustments of current guidelines of moderate hypothermia for asphyxiated newborns might be useful to prevent even more efficiently brain injury in these patients. Such trials are currently ongoing (e.g., “late hypothermia for hypoxic-ischemic encephalopathy,” ClinicalTrials.gov Identifier: NCT00614744), and results should be known in the near future.

One of the main challenges of the current guidelines for cooling newborns with HIE is to initiate the hypothermia within the first 6 hours of birth. In many places around the world, newborns may be born at great distances from neonatal intensive care units offering cooling, preventing them from arriving there within the good timing. Specific protocols have to be developed in collaboration with the cooling centers of reference in order to start cooling safely and reliably prior to the transport at the birthing center and/or during transport, so that these patients can also benefit from this treatment [16, 19, 28]. In particular, continuous measurement of central temperature (esophageal or rectal) should be carefully monitored, in order to target the recommended temperature [16, 19, 28].

## 4. What Are the Potential Alternatives?

Studies of alternative treatments for neonatal HIE are becoming available, with the goal of further increasing the proven benefits of hypothermia, that is, to decrease eventual neonatal brain injury associated with neonatal asphyxia and thus improve future neurodevelopmental outcome. These alternatives include specific brain-oriented therapies, which are targeted to the critical events contributing to tissue damage in neonatal HIE (Table 1) [29]. They usually involve molecules that cross the blood-brain barrier and target mechanisms of injury, which are not (or are not completely) inhibited by hypothermia. These include anticonvulsant or antiexcitatory drugs (e.g., phenobarbital, topiramate, bumetanide, magnesium sulphate, and xenon), antiapoptotic drugs (e.g., erythropoietin), anticalcium drugs (e.g., xenon), or anti-inflammatory or antioxidative drugs (e.g., melatonin and N-acetylcysteine) [30–33]. The goal of these experimental therapies is to produce a synergistic neuroprotective effect with hypothermia [30–33].

Most research efforts in neonatal neurology are currently targeted to neuroprotective strategies, but another concept is slowly emerging from the adult stroke literature. In adults, neurorestorative therapies (Table 1) are currently being developed to treat ischemic brain injury, including cerebral vasculature-based therapy combined with neuroprotection [34], as the more “conventional” strategies (antiapoptosis, anticalcium, anti-inflammation, and antioxidative injury) have been disappointingly ineffective in adult clinical trials. These neurorestorative therapies target the neurovascular unit, composed of functionally integrated cellular (including brain endothelial cells, astrocytes, pericytes and smooth muscle cells, neural stem cells, oligodendrocytes, and neurons) and acellular elements that form the basement membrane [35]. Thus, they lead to enhancement of restorative events, such as endogenous neurogenesis, angiogenesis, axonal sprouting, and synaptogenesis in the ischemic brain, explaining the potential for stimulation of brain plasticity and improvement in functional recovery [36, 37]. In adult models of stroke, these therapies have been shown to activate these processes and successfully reduce the size of the infarcted areas [38, 39] and are now being actively tested in human patients. The immature brain of a newborn is not simply a “small adult brain,” and the mechanisms underlying neonatal HIE is certainly very unique compared to the ones underlying adult stroke [40]. However, evidence is growing in the literature that such a systemic approach might also be useful to better treat neonatal brain injury. One such potential neurorestorative drug in newborns is erythropoietin, which has a recognized role in promoting neural regeneration and neurovascular remodeling [41]. A randomized controlled clinical trial is currently ongoing in human newborns to assess its efficiency (i.e., “neonatal erythropoietin in asphyxiated term newborns,” ClinicalTrials.gov Identifier: NCT00719407). Another promising alternative treatment of neonatal HIE utilizes cord blood and mesenchymal stem cells to replace neurons lost due to brain injury, as well as activate endogenous stem cells, and release growth factors, thus minimizing brain damage and promoting restoration [42, 43].

## 5. What Are the Next Steps before Implementing These Treatments?

These adjunctive therapies have until now mostly been tested only in animal models of HIE or in different populations of newborns (e.g., erythropoietin to reduce the need for blood transfusions in very low birthweight infants [44]). Only a few randomized-controlled studies using these treatments are currently available in human newborns with HIE, and they usually include only small numbers of patients [45]. Before implementation, these alternative therapies will need to prove their efficiency and safety in randomized-controlled clinical trials with appropriate sample size consideration and outcome variables.

Determining the best timing and the best dosage range for these alternative treatments is also crucial to obtain their maximum efficiency. It has been hypothesized that hypothermia may buy additional time for neuroprotective drugs to act within an expanded “therapeutic window” [25]. However, for most of these alternative treatments, it remains to be determined when they will have the maximum synergistic effect with hypothermia treatment. For example, in the first randomized trial of erythropoietin in term newborns with HIE, the medication was given first within the first 24 hours of birth and thereafter every other day for 2 weeks; the results showed a significantly improved primary outcome (death or disability rate) at 18 months, suggesting that this medication may provide therapeutic effect over a longer period than the first 6 hours of life [45]. Further similar trials need to be performed for each of these other potential treatments.

In addition, appropriate selection of the newborns most likely to respond to these adjunctive treatments is of utmost importance to demonstrate more quickly their efficacy. Planning for such trials should include early identification of newborns at risk of developing brain HI injury (whether or not hypothermia is administered). To this end, early biomarkers of future brain HI injury in newborns are required to adjust existing cooling guidelines or to add other therapeutic interventions. Ideally, regular evolution of such biomarkers should be known, so that the impact of these newer treatments may be assessed as soon as possible. Current biomarkers included clinical signs, neurological exam outcomes, and assessment of brain activity by amplitude-integrated electroencephalogram [14, 46, 47]. Recent studies suggest some other potential biomarkers, such as brain magnetic resonance imaging [27], near-infrared spectroscopy [47], or biochemical markers [48], which would help guide the adjustment of therapies. Adjustment of the hypothermia treatment or addition of new treatments should be performed according to the specific clinical and imaging findings of these patients.

## 6. Conclusions

How to treat newborns with HIE following birth asphyxia has become one of today's hottest topics in neonatal medicine. Over the past decade, substantial progress has been made in neurointensive care for newborns. However, much remains to be done to improve these early positive results and reduce further the eventual neonatal brain injury and improve future neurodevelopmental outcome of newborns with a hypoxic-ischemic event at birth. Several promising new treatments for the prevention of the devastating neurological consequences of birth asphyxia are currently being tested, but further detailed animal and clinical studies are required before their safe application in human newborns with HIE.

## 7. Future Research Directions

- Define early biomarkers of brain injury to target the newborns with HIE most vulnerable to brain injury despite hypothermia treatment.
- Determine if adjustments to current guidelines for mild hypothermia (i.e., cool later, lower, or longer) might be useful to further prevent brain injury.
- Determine the best timing and the best dosage range of administration of adjunctive treatments, in order to get their maximum efficiency.
- Study in more detail the synergistic effects of these various treatments to better target the different and specific needs of each asphyxiated newborn infant and decrease the eventual neonatal brain injury and thus improve future neurodevelopmental outcome.
- Perform safety studies in human newborns with HIE for all these potential alternative treatments.

## Figures and Tables

**Table 1:** Potential therapies to treat birth asphyxia in newborns.

*Neuroprotective therapies:*	

Hypothermia	
Drugs that might produce a synergistic neuroprotective effect with hypothermia:	
(i) anticonvulsant or antiexcitatory drugs: for example, phenobarbital, topiramate, bumetanide, magnesium sulphate, xenon	
(ii) anti-apoptotic drugs: for example, erythropoietin	
(iii) anti-calcium drugs: for example, xenon	
(iv) anti-inflammatory or antioxidative drugs: for example, melatonin, N-acetylcysteine	

*Neurorestorative therapies:*	

(i) erythropoietin	
(ii) cord blood and mesenchymal stem cells	

## Abbreviation

HIE: hypoxic-ischemic encephalopathy.
